# Supplementary material for: Identification and functional analysis of non-coding regulatory small RNA FenSr3 in Bacillus amyloliquefaciens LPB-18
Source: PeerJ. 2023 May 15;11:e15236. doi: 10.7717/peerj.15236 (PMC10194069; doi:10.7717/peerj.15236)
Supplement: Supplemental Information 4 [file peerj-11-15236-s004.zip › KO/CK-vs-T1_map/map07024.html]

KEGG PATHWAY: HMG-CoA reductase inhibitors - Reference pathway


|  |  |
| --- | --- |
| **HMG-CoA reductase inhibitors - Reference pathway** |  |

|  |  |  |
| --- | --- | --- |
| [ Pathway menu \| Pathway entry \| Show description \| User data mapping ] | 184% 150% 122% 100% 82% 67% 55% | 图片下载 |

HMG-CoA reductase inhibitors, also known as statins, are a class of cholesterol lowering drugs that target HMG-CoA reductase in the mevalonate pathway. In 1971 Akira Endo and his team working for the Japanese pharmaceutical company Sankyo started a project to search for microbial metabolites that would inhibit HMG-CoA reductase, the rate-limiting enzyme in the cholesterol biosynthesis, hoping that certain microorganisms would produce such inhibitors as a weapon against other microbes. After screening 6,000 microbial strains mevastatin was discovered from Penicillium citrinum, but the clinical trial of mevastatin was not completed because of its toxicity. The first successful statin was lovastatin isolated from Aspergillus terreus by Merck, which had repeated Sankyo's approach to microbial screening. Following the first generation statins that are based on natural products, the second and the third generation statins were developed using chemical synthesis.
